# Supplementary material for: Should we keep some distance from distancing? Regulatory and post-regulatory effects of emotion downregulation
Source: PLoS One. 2021 Sep 2;16(9):e0255800. doi: 10.1371/journal.pone.0255800 (PMC8412372; doi:10.1371/journal.pone.0255800)
Supplement: S1 Fig — (DOCX) [file pone.0255800.s001.docx]

**Diers et al. - Supplementary Material**

Title of the overall project SFB940 A5: *Volitional emotion regulation: The costs of control*

A priori specified design and analysis plan: <http://gepris.dfg.de/gepris/projekt/223659428>;
<https://tu-dresden.de/bereichsuebergreifendes/sfb940/research/a-mechanismen/a5>

Open Materials, Open Data: <https://osf.io/mg5ac/>


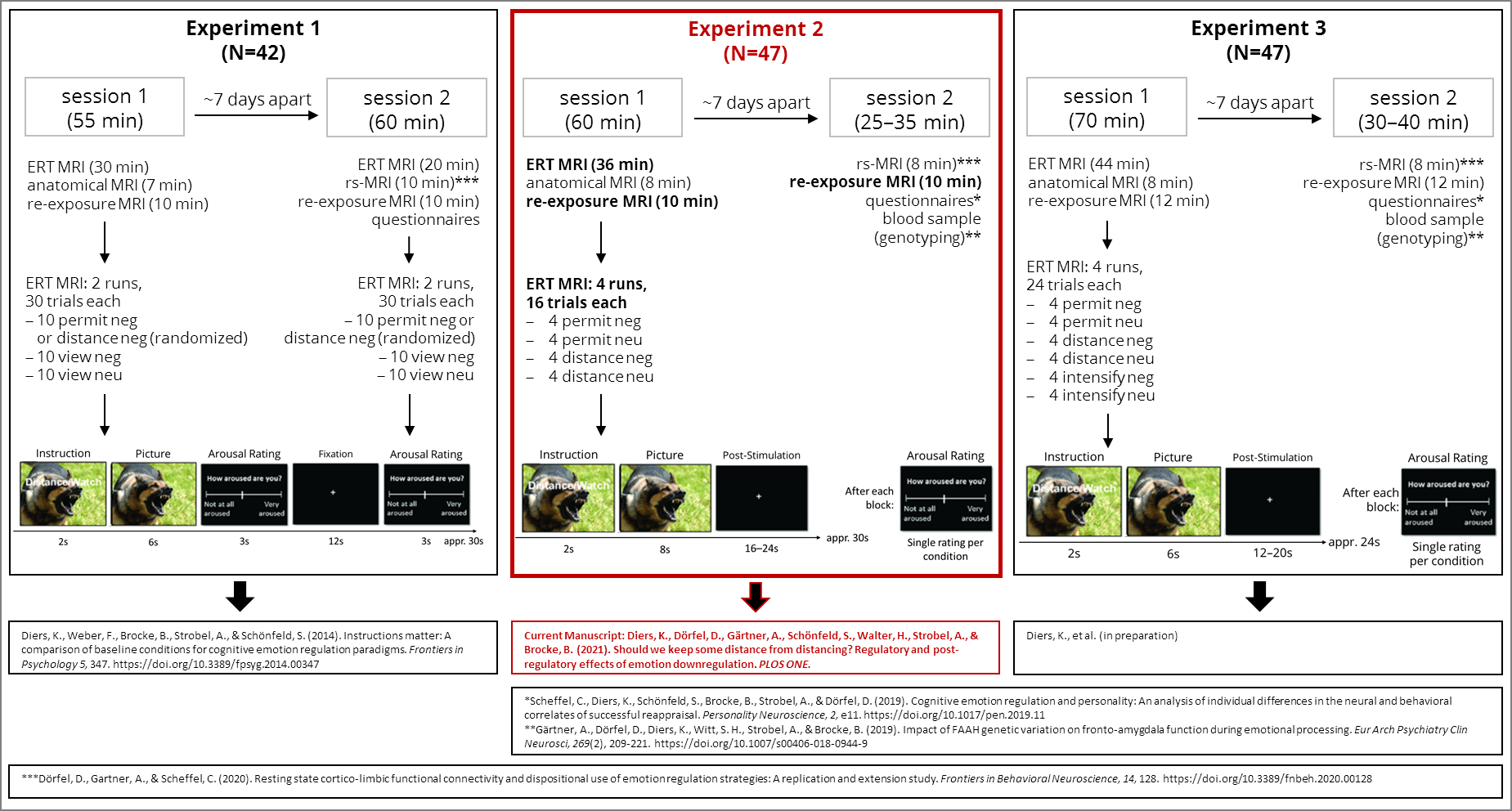


**Fig S1. Overview over the larger project and corresponding peer-reviewed publications and manuscripts, respectively.** The current manuscript is highlighted in red and parts of the experimental procedure relevant for the current manuscript presented in bold letters. ERT = Emotion Regulation Task, rs-MRI = resting-state MRI, neg = negative pictures, neu = neutral pictures,
